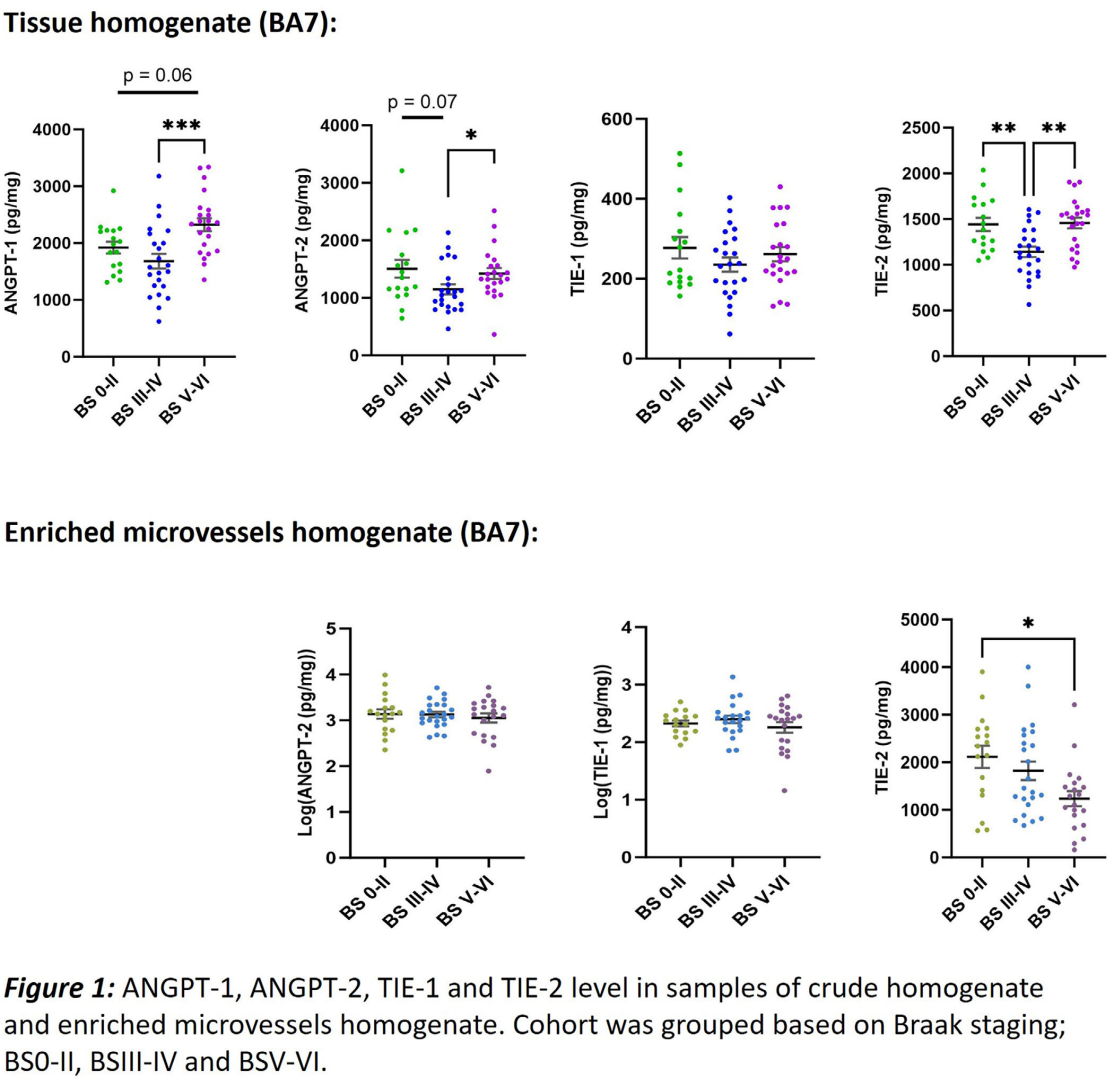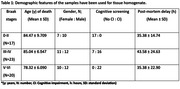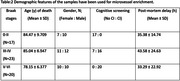# Dysregulated Angiopoietin‐Tie signalling contributes to neurovascular dysfunction in Alzheimer’s disease

**DOI:** 10.1002/alz.095139

**Published:** 2025-01-09

**Authors:** Selvi Ince, Seth Love, James Scott Miners

**Affiliations:** ^1^ University of Bristol, Bristol, Horfield United Kingdom

## Abstract

**Background:**

Cerebral hypoperfusion and blood‐brain barrier (BBB) leakiness within the precuneus is common in early‐stage Alzheimer’s disease (AD). Pericyte‐derived angiopoietin‐1 (ANGPT‐1) activates endothelial TIE‐2 receptors promoting vascular stability whereas endothelial cell‐derived ANGPT‐2, a weak agonist or antagonist of TIE‐2, promotes BBB leakiness. Endothelial TIE‐1 also regulates TIE‐2 signalling. We recently showed elevated ANGPT2 level in mild‐cognitive impairment related to albumin, a marker of BBB leakiness, in CSF. In this study, we examined ANGPT/TIE signalling in brain tissue and microvessel‐enriched fractions (MVFs) in relation to AD progression and cerebrovascular injury.

**Method:**

We studied parietal cortex (Brodmann area 7) from Braak tangle stage (BS) 0‐II (n = 17), III‐IV (n = 23) and V‐VI (n = 22) from cases obtained from the South‐West Dementia Brain Bank, University of Bristol. ANGPT‐1, ANGPT‐2, TIE‐1, and TIE‐2 levels in brain tissue and MVFs were measured by ELISA. CD31 level was used to adjust for endothelial content. MAG and PLP1 levels, to assess brain oxygenation and fibrinogen, to assess BBB integrity, were measured by ELISA. One‐way ANOVA with Tukey’s post hoc testing was used to compare groups and Pearson’s correlation coefficient was used to assess relationships between ANGPT/TIE, MAG:PLP and fibrinogen.

**Result:**

In crude homogenates, ANGPT‐1 level was elevated in BSV‐VI compared to BS0‐II (p = 0.06) and BSIII‐IV (p = 0.0005). TIE‐2 was reduced in BSIII‐IV compared to BS0‐II (p = 0.004) and BSV‐VI (p = 0.001) in crude homogenates and was lower in MVFs in BSV‐VI than BS0‐II (p = 0.02). ANGPT‐2 level was higher in BSV‐VI than BSIII‐IV in brain tissue (p = 0.04). However, both ANGPT‐2 and TIE‐1 levels were unaltered in relation to BS in MVFs. Across the entire cohort, ANGPT‐2 level correlated inversely with MAG:PLP1 ratio in BSIII‐IV (p = 0.052, r = ‐0.42) but positively with MAG:PLP1 (p = 0.04, r = 0.46) and fibrinogen level (p = 0.02, r = 0.49) in BSV‐VI brains.

**Conclusion:**

The expression of the stabilising receptor, TIE‐2, was reduced in early‐stage AD and ANGPT2 expression was differentially related to perfusion and BBB leakiness dependent on disease stage. The study highlights important differences in ANGPT/TIE signalling in relation to disease stage and illustrates a complex relationship between brain, microvascular, and CSF changes that warrants further investigation.